# Distinguishing the Attentional Mechanisms of Distinct Mindfulness States: A Computational Modeling Comparison of Focused Attention and Open Monitoring

**DOI:** 10.1007/s12671-026-02781-2

**Published:** 2026-03-09

**Authors:** Yanli Lin, Grant S. Shields

**Affiliations:** Department of Psychological Science, University of Arkansas at Fayetteville, Fayetteville, United States

**Keywords:** Mindfulness, Attention, Attentional control, Shrinking spotlight, Focused attention, Open monitoring

## Abstract

**Objectives:**

Focused attention (FA) and open monitoring (OM) meditation are theorized to confer distinct neurobehavioral influences, yet the specific cognitive mechanisms underlying these effects remain poorly understood. This study leveraged computational modeling to formally test and distinguish how these two mindfulness states modulate theoretical processes of attention control.

**Method:**

We analyzed flanker task data from a prior study in which 29 meditation-naïve participants completed a fully within-subject crossover protocol, involving brief state inductions of FA, OM, and active control. We then fit a shrinking spotlight (SSP) computational model to quantify parameters of attentional scope, decision thresholds, and nondecision-related processing.

**Results:**

As hypothesized, FA decreased the maximum “width” of the attentional spotlight compared to OM and control conditions (all *t* >|8.43|, all *p* < 0.001), while slowing the rate of narrowing (all *t* >|7.29|, all *p* < 0.001). In contrast, OM accelerated the rate of attention narrowing (*t*(82) = 2.18, *p* = 0.032) and increased nondecision time (all *t* > 2.01, all *p* < 0.048).

**Conclusions:**

These results indicate that FA narrows attentional scope, possibly reducing initial distraction but with limitations to rapidly recalibrate attention beyond the initial scope of focus. In contrast, OM appears to improve the speed of selecting relevant targets, while slowing perceptual-motor encoding and motor execution. Together, these findings provide model-based evidence for the distinct attentional mechanisms of FA and OM and illustrate the utility of computational cognitive modeling for testing key theories within mindfulness science.

**Preregistration:**

This study was not preregistered.

Scientific interest in mindfulness, broadly defined as intentional nonjudgmental awareness of present momentary experience (Bishop et al., 2004; Kabat-Zinn, 1990), has surged in recent decades, with a significant portion of investigative enthusiasm centering on how mindfulness training might influence, and potentially benefit, fundamental cognitive functions (e.g., Chiesa et al., 2011; Lin et al., 2022; Whitfield et al., 2022). Central to the practice of mindfulness are two distinct, yet often complementary, meditation techniques: focused attention (FA) and open monitoring (OM). Briefly, FA involves deliberately sustaining attention on a chosen target object (e.g., the breath), with continuous redirection whenever mind wandering is detected, whereas OM cultivates open non-reactive monitoring of mental and sensory experiences (e.g., thoughts, feelings, physical sensations) as they naturally arise (Britton et al., 2018; Lutz et al., 2008, 2015). Although these practices have become increasingly well differentiated from both theoretical and empirical perspectives (Brown et al., 2022; Fox et al., 2016; Lin et al., 2024a, b; Lohani et al., 2020; Manna et al., 2010), the precise mechanisms through which FA and OM exert their cognitive effects remain poorly understood and in need of further investigation.

A key limitation maintaining this knowledge gap is that traditional behavioral measures (e.g., accuracy, reaction time), while informative for assessing mindfulness *effects* on various domains of cognition, often fail to adequately capture the underlying *processes* that give rise to observed behavior. Aimed directly at remediating such problems, computational modeling offers a powerful method to formalize mechanistic theories of cognition into mathematical expressions, enabling latent parameter estimation to quantify the distinct contributions of specific cognitive processes (e.g., evidence accumulation, decision boundaries, attention allocation) on task performance (Farrell & Lewandowsky, 2018; Ratcliff & McKoon, 2008; Ratcliff et al., 2016). In light of these advantages, there have been explicit calls (see van Vugt et al., 2019) and increasing interest in harnessing the significant potential of computational modeling within the context of mindfulness research. Consequently, various drift-diffusion models (DDM) for example, have been applied to investigate how mindfulness modulates key decision-making parameters such as decision thresholds, evidence accumulation rate, and learning (Golubickis et al., 2024; van Vugt & Jha, 2011; van Vugt & van den Hurk, 2017). Although these studies showcase the utility of the approach, applying computational modeling to advance the cognitive science of mindfulness, including differentiation of FA and OM, remains in its earliest stages, with many investigative possibilities and promising models left unexplored.

Indeed, although DDM is useful for probing decision-level processes, the key theoretical distinctions between FA and OM often center around the scope and object of attention (Lutz et al., 2008, 2015). Therefore, alternative models designed specifically to assess attentional processes may prove exceptionally fruitful toward distinguishing the cognitive mechanisms of FA and OM. One promising possibility is the shrinking spotlight, a class of models that conceptualizes attention as a visuospatial spotlight which can vary in scope and processing intensity (White & Curl, 2018; White et al., 2011). A narrower spotlight is theorized to enhance information processing at the attended location but may fail to capture peripheral items, while a broader spotlight can hold more information simultaneously, though at the potential expense of diminished processing resolution for any subset of attended items. Critically, these defining characteristics of the shrinking spotlight model align remarkably well with the hypothesized attentional properties of FA and OM. FA, with its namesake emphasis on sustained focus, has been theorized to cultivate a narrower scope of attention, possibly leading to enhanced processing of target stimuli while decreasing the response potency of distractors (Lutz et al., 2008, 2015; Ullrich et al., 2021). Conversely, OM, characterized by open awareness, is thought to foster a wider attentional spotlight, allowing for more diffuse processing of a broader range of internal and external stimuli. Despite the natural linkage and clear conceptual overlap, to our knowledge, the shrinking spotlight or other related computational models have yet to be implemented to empirically test the putative differences in visual attention processing between FA and OM.

The present study aimed to address this missed opportunity by fitting an adapted version of the shrinking spotlight model to flanker task data collected from a recently completed study that directly manipulated and compared the neurobehavioral effects of FA and OM using a fully within-subject state induction design (Lin et al., 2024a). Briefly, the parent study found that OM selectively induced a more cautious and intentional response style, characterized by higher accuracy, slower reaction times (RTs), and reduced P3 amplitude, whereas FA did not produce the hypothesized reduction in flanker interference effects. Although these results illustrate the neurocognitive distinctions between FA and OM, computational modeling approaches may be better suited to capture the subtle attentional processes that may further distinguish FA vs. OM effects on task performance. Consequently, by fitting the shrinking spotlight model to the data, we sought to elucidate for the first time how these distinct mindfulness states may differentially modulate the dynamics of attentional scope and information processing.

Expanding upon the conceptual framework outlined above, we hypothesized that the FA state, relative to OM and an active control condition, would be selectively characterized by model parameters indicative of a narrower attentional spotlight; in contrast, we hypothesized that the OM state would be uniquely associated with parameters suggestive of a broader attentional spotlight coupled with greater response caution. Ultimately, by elucidating the specific attention processes modulated by FA and OM, the current study aims to directly test long-held theoretical distinctions between these foundational mindfulness states and practices, while simultaneously advancing the application of computational modeling approaches toward understanding mindfulness effects on human cognition.

## Method

### Participants

Thirty healthy, mindfulness-naïve, fluent or native English-speaking participants were recruited and enrolled in the study. As reported previously, one participant was excluded from all analyses due to repeated failure to comply with task instructions, resulting in a final sample of twenty-nine participants (17 females, 12 males, *M*_age_ = 20.72 years, SD_age_ = 4.04 years). Moreover, two sessions of data (one each from two participants) were removed due to poor accuracy (3 SD below group mean) based on preregistered outlier exclusion criteria. Importantly, the data used here for computational modeling were identical to the parent study (Lin et al., 2024a), which provides a complete description of the recruitment procedures, sample characteristics, and inclusion/exclusion criteria. All data, materials, and analysis code from the original study are publicly available via that publication; specific code and output for the current analyses are available via the Open Science Framework (https://osf.io/uv9yn/).

### Procedures

Briefly, the study utilized a fully within-subject state induction protocol, during which each participant completed three laboratory testing sessions occurring across separate days (all completed within 1 week of beginning the first session). Each session involved either the FA, OM, or active control (C) induction, with the order of sessions randomized across participants to minimize order effects.

At the beginning of each FA/OM session, participants listened to a 10-min guided audio recording designed to induce the respective mindfulness state, followed by active instructions to maintain the FA/OM state during task performance. Each audio induction was administered twice per session, once immediately before the flanker task and another before an affective picture viewing task (task order randomized), to help participants reacquire and sustain the intended state. Moreover, participants were explicitly reminded to maintain the cultivated FA or OM state immediately prior to performing the task. In the C session, participants listened to a 10-min duration-matched educational TED talk, followed by non-specific instructions for approaching the tasks. Analytic and procedural details pertaining to the affective picture viewing task have been reported elsewhere (Lin et al., 2024b); these details will not be discussed further here.

A manipulation check questionnaire (reported in Lin et al., 2024a), assessed participant engagement and responsivity during both the induction and task performance periods. For the audio inductions, participants rated the control TED talk slightly more positively, engaging, and comfortable than both guided meditations but reported higher comprehension for FA and comparable levels of interest and arousal across FA and OM. Critically, for the flanker task, no differences emerged across conditions, including engagement, difficulty, arousal, and sleepiness, with the sole exception that participants reported that the task was more interesting following OM relative to control. Collectively, these data indicate that participants remained similarly engaged during flanker performance across induction conditions and did not report notable difficulty understanding or maintaining instructions.

### Measures

#### Tasks

##### Audio Inductions

To maintain standardization, both mindfulness inductions were recorded by a certified MBSR teacher. The FA induction guided participants to sustain attention on their breath and to redirect focus back to the breath whenever mind wandering was detected, whereas the OM induction fostered open, nonjudgmental awareness of arising thoughts, feelings, or physical sensations. The active control C induction was a condensed audio recording of a TED talk on how to learn a second language quickly by the linguist Chris Lonsdale. All three induction recordings were exactly 10 min in duration and have been successfully implemented in prior work (Lin et al., 2019; Tang & Braver, 2020). Finally, participants were instructed to keep their eyes open during the audio inductions based on prior evidence that mindfulness inductions may selectively promote sleepiness during eyes-closed conditions among novices (Lin et al., 2020).

##### Flanker Task

The study administered an arrow version of the Eriksen flanker task (Eriksen & Eriksen, 1974). Briefly, participants were presented with a five-arrow array that was either directionally congruent (e.g., >  >  >  > >) or incongruent (e.g., >  >  <  > >) with the center arrow. Participants were instructed to respond as quickly and accurately to the direction of the target central arrow by pressing the respective mouse button using their right index or middle finger. Each stimulus array was displayed for 200 ms, followed by a 950-ms response window. The intertrial interval varied randomly between 600 and 1000 ms. Participants completed a total of 512 trials, evenly divided between congruent and incongruent trials, across 8 blocks of 64 trials. The task was programmed and presented using E-Prime software (Psychology Software Tools Inc, Sharpsburg, PA, USA).

### Data Analyses

#### Computational Model Specification

In line with many models of executive attention and control, the shrinking spotlight (SSP; White et al., 2011) posits that attentional breadth begins wide and progressively narrows onto the goal-relevant stimulus. Formally, this initial attentional breadth (sd_*a*(0)_) and its narrowing (*r*_*d*_) are modeled via a linear decrease:

$$sd_{a\left(t\right)}=sd_{a\left(t-1\right)}-r_{d\left(t\right)}$$

Target and flanking stimuli are assumed to have a unit width of 1.0, with both attention and the target centered at 0.0. Activations of inner and outer stimuli are estimated by the following equations, where *Φ* denotes the cumulative distribution function of the normal distribution, which sum to 1:

$${a}_{\mathrm{outer}-\text{right }(t)}= {\int }_{1.5}^{inf}\phi \left(0, {\mathrm{sd}}_{a \left(t\right)}\right)$$

$${a}_{\mathrm{inner}-\mathrm{right} (t)}= {\int }_{0.5}^{1.5}\phi \left(0, {\mathrm{sd}}_{a \left(t\right)}\right)$$

$${a}_{\mathrm{target} (t)}= {\int }_{-0.5}^{0.5}\phi \left(0, {\mathrm{sd}}_{a \left(t\right)}\right)$$

$${a}_{\mathrm{inner}-\mathrm{left} (t)}= {\int }_{-1.5}^{-0.5}\phi \left(0, {\mathrm{sd}}_{a \left(t\right)}\right)$$

$${a}_{\mathrm{outer}-\mathrm{left} (t)}= {\int }_{\mathrm{inf}}^{-1.5}\phi \left(0, {\mathrm{sd}}_{a \left(t\right)}\right)$$

Finally, the mean drift of the evidence accumulation diffusion process [i.e., *X*_*t*_ = *X*_*t−*1_ + *N*(*v*_*t*_, *σ*)] (*σ* is fixed because drift, threshold, and *σ* are not concurrently identifiable; it was fixed to 7.0 in this study) is modeled as the sum of all stimuli multiplied by estimated perceptual input parameter *P*. Because drift, threshold, and *σ* are not concurrently identifiable without other forms of data, *σ* functions as a scaling constant whose value is arbitrary because values of drift and boundary scale make inferences identical across values of *σ* (Nunez et al., 2025). We chose to use 7.0 because, with RTs in milliseconds rather than seconds (as was used in White et al., 2011), preliminary modeling suggested that a *σ* of 7 appeared to produce similar inferences for values of *P* and boundary that were described in White et al. (2011).

$${v}_{(t)}= \sum_{i}^{N a}P\times {a}_{i (t)}$$

In psychological terms, the perceptual input parameter represents the strength of the sensory evidence entering the decision process. In other words, it indexes how effectively attention amplifies task-relevant visual information, with higher values corresponding to stronger perceptual gain for attended stimuli. As shown in the above, goal-irrelevant stimuli (e.g., flanking arrows) contribute to response selection to the extent that they are within the attentional window, which is greater during the earlier portion of a trial. The sign of *P* is positive for all nontarget *a* on congruent trials and negative on incongruent trials.

In addition to fitting parameters for attentional processes, the model also fits parameters for the time that it takes to encode and execute a chosen motor action (i.e., nondecision time, *Ndt*), variability in the time it takes to encode and execute a chosen motor action (i.e., nondecision time variability, *Ndtσ*), and the amount of information needed before making a decision (i.e., decision boundary, *b*).

#### Parameter Estimation

To fit the model to trial data, the model was fit to cumulative density functions (CDFs), capturing response time distributions (0.1, 0.3, 0.5, 0.7, 0.9, 1 sextiles), and conditional accuracy functions (CAFs; 0.25, 0.5, 0.75, 1.0 quartiles), which constitute the error data considered in the fitting procedure (Servant et al., 2016; White & Curl, 2018). Parameters were constrained to be positive numbers. The observed and predicted CDFs and CAFs were then fit by minimizing the log likelihood of summed −2 log binomial probability densities of each cell corresponding to the cell’s model predictions. For determining goodness of fit post-fitting, we calculated a chi square statistic:

$${\chi }^{2}={N}_{i} \frac{{({p}_{ij}- {\pi }_{ij})}^{2}}{{\pi }_{ij}}$$

where *p*_*ij*_ represents the observed and π_*ij*_ represents the predicted proportion of trials in bin *j* of trial type (i.e., congruent, incongruent) *i*, and *N*_*i*_ represents the number of trials per trial type *i*.

We fit the model in Julia, using the Nelder-Mead simplex method via the following hierarchical procedure. First, we estimated a single set of population-level parameters by fitting the model to all data across all participants. We generated 100 parameter values for [sd_*a*(0)_, *r*_*d*_, *P*, *b*, *Ndt*, *Ndtσ*] by drawing from truncated normal distributions. These distributions were selected to ensure that the parameters had mean values and weak priors (i.e., large SDs relative to bounds) from previous work (White et al., 2011), and that the parameters were within bounds that would produce reaction time and error distributions that had bare minimum plausibility (e.g., distributions were not entirely errors). Specifically, distributions had means [1.8, 0.017, 0.6, 60, 250, 30] and standard deviations [1.2, 0.05, 0.3, 30, 60, 20], lower bounds [0.8, 0.005, 0.2, 20, 175, 10], and upper bounds [4.0, 0.15, 2.0, 150, 375, 50]. We simulated 25,000 trials (12,500 congruent, 12,500 incongruent) for each conjunction of parameters during each fitting step. The best-fitting parameters from these 100 minimization routines were saved as the population parameter values.

Second, we fit deviation (*Δ*) parameters around the population parameters corresponding to each condition’s deviation. We generated 100 parameter sets for each of the three conditions (300 parameter sets total) by drawing from truncated normal distributions with means of 0 and standard deviations [1.2, 0.05, 0.3, 30, 60, 20]. The bounds of these truncated normal distributions were constrained such that population + condition values did not fall below 0 (lower bounds) or above [6.0, 0.2, 5.0, 200, 450, 90] (upper bounds). Each minimization routine only included data from one of the three conditions (across participants). The best-fitting parameters from these 100 minimization routines for each condition were saved as that condition’s deviation (*Δ*) parameter values.

Third, and independent of condition, we fit deviation (*Δ*) parameters around the population means corresponding to each participant. We generated 100 parameter sets for each of the 29 participants (2900 parameter sets total) by drawing from truncated normal distributions with means of 0 and standard deviations [1.2, 0.05, 0.3, 30, 60, 20]. The bounds of these truncated normal distributions were constrained such that population + participant values did not fall below 0 (lower bounds) or above [6.0, 0.2, 5, 200, 450, 90] (upper bounds). Each minimization routine only included data from the one participant (across conditions). The best-fitting parameters from these 100 minimization routines for each condition were saved as that participant’s deviation (*Δ*) values.

Because the participant and condition parameter deviations around the population are independently estimated maximum likelihood estimates, concurrently summing these deviations with population parameters produces, for each parameter, a value that varies from the true parameter value only by observation variance—a nondifferentiable conjunction of participant × condition variance and error variance. This sum has two desirable properties, which we leverage for analyses below. First, when analyzed in a general linear model, this approach removes the same variance as that which is removed by a repeated measures ANOVA or MANOVA, with the critical difference that this approach, unlike a repeated measures ANOVA, does not require observation-level estimates, and thus is not limited by parameter bounds and resultant truncation that is required for estimating parameters at the level of observation. Although the values that fall outside of parameter boundaries make producing graphs of expected values difficult, they are more suited to analysis of within statistical models that assume normal (e.g., nontruncated) distributions, such as ANOVAs and linear models. Second, this approach makes explicit the hierarchical structure of the data. Parameter estimation at the level of observation cannot differentiate error variance from true variance, and can thus produce estimates biased by error (Farrell & Lewandowsky, 2018). Estimating population parameter values by fitting all participants’ data sequentially (non-aggregated) to a single set of parameters, then estimating condition deviation parameters by fitting every participants’ data within that condition to that condition’s deviation parameters, and concurrently estimating participant deviation parameters by fitting every condition’s data within that participant to that participant’s deviation parameters ensures that the resultant sum respects the hierarchical structure of the data and does not fit error variance as true variance. As will be described below, results were largely consistent when we analyzed the observation-level estimates. We refer to regressions using the population +* Δ*condition +* Δ*participant estimates as within-transformed variable regressions.

Finally, for estimation of model fit, considering both condition and participant deviations (*Δ*), we fit deviation parameters around parameter population + *Δ*condition + *Δ*participant values, corresponding to each observation’s noise *Δ*. This sum is equivalent to an observation-level estimation that is weakly informed by the data’s hierarchical structure. We generated 100 parameter sets for each of the 85 observations (8500 parameter sets total) by drawing from truncated normal distributions with means of 0 and standard deviations [1.2, 0.05, 0.3, 30, 60, 20]. The bounds of these truncated normal distributions were constrained such that population + *Δ*condition + *Δ*participant + *Δ*noise values did not fall below 0 (lower bounds) or above [6.0, 0.2, 5.0, 200, 450, 90] (upper bounds). Each minimization routine only included data from the one participant-by-condition observation. The best-fitting parameters from these 100 minimization routines for each observation were saved as that participant-by-condition noise deviation. These final best-fitting parameters—population + *Δ*condition + *Δ*participant + *Δ*noise—were saved as each observation’s parameter values. We refer to these sums as observation-level estimates because they represent the lowest level of estimation (the participant-by-condition level), capturing the nondifferentiable conjunction of participant, condition, and error variance, all constrained to the model’s upper and lower bounds.

The key model parameter estimates for each participant in each of the three conditions were (1) attention spotlight width; (2) shrinkage rate; (3) mean nondecision time; (4) boundary separation; and (5) perceptual input.

#### Statistical Analyses and Hypothesis Testing

Model parameters were derived as described above. For analyses of model fit, we used the observation-level estimates to quantify fit to the data at each observation. We analyzed fit statistics using a multivariate ANOVA (MANOVA), with induction (FA, OM, C) as a within-subjects factor and the model fit statistic (*χ*^2^) as the dependent variable. Analyses of observation-level parameters took the same approach (i.e., ANOVA), with induction (FA, OM, C) as a within-subjects factor and the model parameters (sd_*a*(0)_, *r*_*d*_, *P*, *b*, *Ndt*, *Ndtσ*) as dependent variables. For the hierarchically estimated parameter analyses, because those estimates effectively denoise data similar to multivariate analyses for repeated measures, we used a general linear model and set induction condition as a fixed effect. When significant effects emerged, post hoc pairwise comparisons between conditions were performed to parse the specific pattern of differences. Nonsignificant results were treated as inconclusive rather than as evidence of equivalence, and were thus reported accordingly (i.e., that no statistically reliable difference was detected in the sample). Nonetheless, we also report Bayes factor analyses alongside each ANOVA. By convention, a Bayes factor BF_10_ > 3.0 indicates substantial support in favor of the alternative hypothesis, and a Bayes factor BF_10_ < 0.33 indicates substantial support in favor of the null hypothesis. ANOVAs were conducted using the R package afex, post hoc tests were performed on estimated marginal means using the R package emmeans (which also produced the pairwise effect sizes via eff_size), and Bayes factor analyses were conducted using the R packages BayesFactor. It is important to note that although follow-up tests on frequentist statistics used estimated marginal means and their standard errors (via the emmeans package), the Bayes factors for those follow-up tests were conducted on the raw data described for each test (i.e., ttestBF package uses the original data, not the fits from the model that produced the *F* value).

The above allowed us to test the primary hypothesis—namely, that FA and OM will confer differentiable effects on attentional spotlight width and decision-making. Consistent with the aforementioned rationale, we specifically predicted that FA would be characterized by a narrower attentional spotlight as evidenced by a significantly smaller spotlight width parameter relative to both OM and C. Conversely, we expected that the OM induction would produce larger width and boundary separation estimates compared to FA and C, collectively suggestive of a wider attentional spotlight coupled with higher evidence threshold.

## Results

A comprehensive analysis of the behavioral and electrophysiological data from this study is detailed in the parent publication (Lin et al., 2024a); here, we provide a concise summary of the behavioral findings to contextualize the present modeling results. In brief, OM was associated with higher accuracy and slower response times relative to both FA and the active control condition, consistent with a more deliberate or cautious response strategy, whereas FA did not significantly alter flanker performance across accuracy or response times. Importantly, no condition differences emerged in perceived task engagement or difficulty, indicating comparable compliance across task sessions. In the sections that follow, we focus on reporting the computational modeling results, beginning with model fit indices, followed by the effects of the mindfulness inductions on estimated model parameters.

### Model Fit

**Fig. 1:**
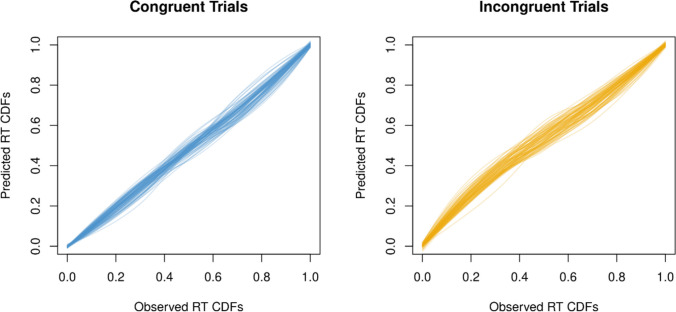
Predicted and observed response time cumulative distribution functions. *Note*. *RT* = response time, *CDF* = cumulative distribution function. Predicted and observed RT CDFs are separated across congruent (left) and incongruent (right) trials. Close correspondence between predicted and observed CDFs indicates good model fit

**Fig. 2:**
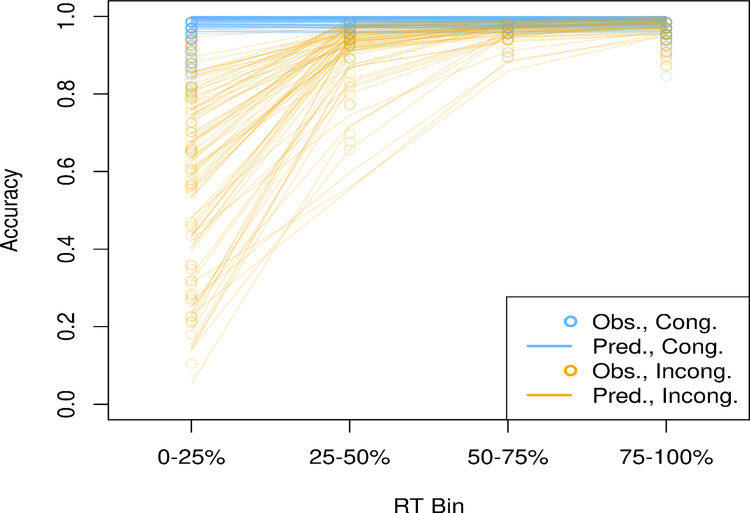
Predicted and observed conditional accuracy functions. *Note*. *RT* = response time. Predicted and observed conditional accuracy functions (CAFs) for congruent (blue) and incongruent (orange). Accuracy is plotted across RT quartile bins. Circles represent observed accuracy, whereas lines reflect model predicted accuracy. Close correspondence between predicted and observed CAFs indicates good model fit

We first assessed the fit of the model to the data, both at the participant level and at the group level. Aggregated across individually fitted datasets and observations, the model was a good fit to the data, with root mean square error of approximation (RMSEA) = 0.005 (RMSEA < 0.05 indicates acceptable fit). At the level of individual participant-by-condition, the model provided an acceptable fit to 82.4% of datasets. Specifically, an acceptable fit was defined as a nonsignificant *χ*^2^ statistic indicating no significant misfit within that dataset. Importantly, fit did not differ as a function of meditation condition, *F*(2, 52) = 0.52, *p* = 0.595, *η*^2^_*p*_ = 0.020, BF_10_ = 0.19, indicating that differences in parameter estimates by condition are unlikely attributable to differences in model fit. Predicted and observed response time data are depicted in Fig. 1, and predicted and observed accuracy data are depicted in Fig. 2.

### Induction Effects on Model Parameters

Consistent with our primary hypothesis, we found a significant effect of induction condition on the initial attentional spotlight width parameter *sd*_a__(0)_,*F*(2, 82) = 49.35, *p* < 0.001, *η*^2^_*p*_ = 0.546, BF_10_ > 1000. Follow-up pairwise comparisons revealed that the FA induction (*M* = 0.42, *SE* = 0.34) produced a significantly narrower initial attentional spotlight than that seen in both the OM condition (*M* = 4.63, *SE* = 0.33), *t*(82) = −8.86, *p* < 0.001, *d* = −1.62, BF_10_ > 1000, and the active control condition (*M* = 4.43, *SE* = 0.33), *t*(82) = −8.43, *p* < 0.001, *d* = −1.54, BF_10_ > 1000. Conversely, and failing to support our second hypothesis, no statistically reliable difference in initial spotlight width was detected between the OM induction condition and the active control condition in this sample, *t*(82) = −0.44, *p* = 0.664, *d* = −0.08, BF_10_ = 0.29.

We also observed a significant effect of induction condition on the shrinkage rate parameter *r*_*d*_, *F*(2, 82) = 48.37, *p* < 0.001, *η*^2^_*p*_ = 0.541, BF_10_ > 1000. This effect was largely (though, this time, not exclusively) driven by the FA induction (*M* = 0.001, *SE* = 0.008), which showed a slower rate of attentional narrowing than both the OM condition (*M* = 0.105, *SE* = 0.008), *t*(82) = −9.43, *p* < 0.001, *d* = −1.73, BF_10_ > 1000, and the active control condition (*M* = 0.081, *SE* = 0.008), *t*(82) = −7.29, *p* < 0.001, *d* = −1.34, BF_10_ > 1000. In contrast, the OM condition showed a faster rate of attentional narrowing than the active control condition, *t*(82) = 2.18, *p* = 0.032, *d* = 0.39, BF_10_ = 1.94.

Additionally, we observed a marginal effect of induction condition on mean nondecision time *Ndt*, *F*(2, 82) = 3.09, *p* = 0.051, *η*^2^_*p*_ = 0.070, BF_10_ = 1.15. This effect was driven by the OM induction (*M* = 285.0, *SE* = 4.2), which showed a greater mean nondecision time than both the FA induction (*M* = 271.2, *SE* = 4.4), *t*(82) = 2.26, *p* = 0.027, *d* = 0.59, BF_10_ = 3.37, and the active control condition (*M* = 273.0, *SE* = 4.2), *t*(82) = 2.01, *p* = 0.048, *d* = 0.51, BF_10_ = 1.39. No statistically reliable difference in mean nondecision time was detected between the FA condition and active control, *t*(82) = −0.29, *p* = 0.773, *d* = −0.08, BF_10_ = 0.31.

Inconsistent with our hypothesis that the OM induction would elevate response caution, there was no significant main effect of induction condition on the boundary separation parameter *b*, *F*(2, 82) = 0.49, *p* = 0.617, *η*^2^_*p*_ = 0.012, BF_10_ = 0.15. Additionally, although the active control condition showed numerically weaker strength of perceptual input than either meditation induction condition, no statistically reliable effect of induction condition was detected on the strength of perceptual input parameter *P*, *F*(2, 82) = 1.28, *p* = 0.283, *η*^2^_*p*_ = 0.030, BF_10_ = 0.28.

Additionally, although not a primary parameter estimate of interest, we observed a significant effect of induction condition on variability in nondecision time, *Ndtσ*, *F*(2, 82) = 14.80, *p* < 0.001, *η*^2^_*p*_ = 0.265, BF_10_ > 1000. Similar to the mean nondecision time results above, this effect was driven by an effect of the OM induction (*M* = 31.42, *SE* = 1.98), which showed greater nondecision time variability than both the FA induction (*M* = 17.65, *SE* = 2.05), *t*(82) = 4.83, *p* < 0.001, *d* = 1.12, BF_10_ = 767.96, and the active control condition (*M* = 18.64, *SE* = 1.98), *t*(82) = 4.56, *p* < 0.001, *d* = 1.04, BF_10_ = 753.37. No statistically reliable FA vs. active control difference in nondecision time variability was detected in this sample, *t*(82) = −0.45, *p* = 0.729, *d* = −0.08, BF_10_ = 0.29.

Finally, following White and Curl, 2018), we ran a sensitivity analysis on the ratio of *sd*_*a*(0)_ to *r*_*d*_, (i.e., *sd*_*a*(0)_/*r*_*d*_). Because sd_*a*_ and *r*_*d*_ are often correlated (and indeed in this sample *r* = 0.83), this ratio serves as a “trade-invariant” index that, reflects the time in milliseconds until the minimum attentional window possible (i.e., *sd*_*a*_ = 0.001) is reached. We observed a significant effect of induction condition on *sd*_*a*(0)_/*r*_*d*_, *F*(2, 82) = 9.25, *p* < 0.001, *η*^2^_*p*_ = 0.184, BF_10_ = 114.28. This effect was driven by graded induction condition differences, with the OM induction (*M* = 43.8, *SE* = 1.6), which showed the lowest *sd*_*a*(0)_/*r*_*d*_ ratio, lower than both the FA induction (*M* = 53.6, *SE* = 1.7), *t*(82) = 4.26, *p* < 0.001, *d* = 1.04, BF_10_ = 342.76, and the active control condition (*M* = 49.8, *SE* = 1.6), *t*(82) = 2.64, *p* = 0.010, *d* = 0.63, BF_10_ = 100.67. Paralleling the slower rate of decrease results, the FA induction condition showed a marginally larger *sd*_*a*(0)_/*r*_*d*_ ratio than the active control condition, *t*(82) = 1.67, *p* = 0.099, *d* = 0.41, BF_10_ = 0.86. Critically, these condition differences indicate that the parameters are not merely trading off to produce equivalent ratios; but rather they reflect distinct functional profiles, wherein FA maintains a narrower spotlight with slower shrinkage, whereas OM is characterized by the fastest and possibly more efficient convergence of attention.

#### Sensitivity Analyses: Observation-Level Parameter Estimates

To comparatively contextualize the findings from our hierarchical analysis, we conducted a sensitivity analysis using a more standard method based on observation-level estimates. Although common, this traditional approach relies on observation-level variance, as the nondifferentiable conjunction of participant × condition and error, rendering it susceptible to overfitting error variance. The results from this analysis largely replicated our primary findings for attentional parameters, but they differed for nondecision time parameters and the *sd*_*a*(0)_/*r*_*d*_ ratio.

Specifically, we again found a significant main effect of induction condition on the attentional spotlight width parameter *sd*_*a*(0)_, *F*(2, 52) = 8.22, *p* < 0.001, *η*^2^_*p*_ = 0.240, BF_10_ = 62.85. Post hoc pairwise comparisons revealed that the FA induction produced a significantly narrower attentional spotlight (*M* = 2.14, *SE* = 0.26) relative to both the OM (*M* = 3.61, *SE* = 0.40), *t*(26) = −3.12, *p* = 0.004, *d* = −0.74, BF_10_ = 9.32, and active control (*M* = 3.55, *SE* = 0.40) conditions, *t*(26) = −3.23, *p* = 0.003, *d* = −0.71, BF_10_ = 11.87. Conversely, no statistically reliable difference in spotlight width was observed between OM and the active control condition, *t*(26) = −0.19, *p* = 0.856, *d* = −0.03, BF_10_ = 0.20.

**Fig. 3:**
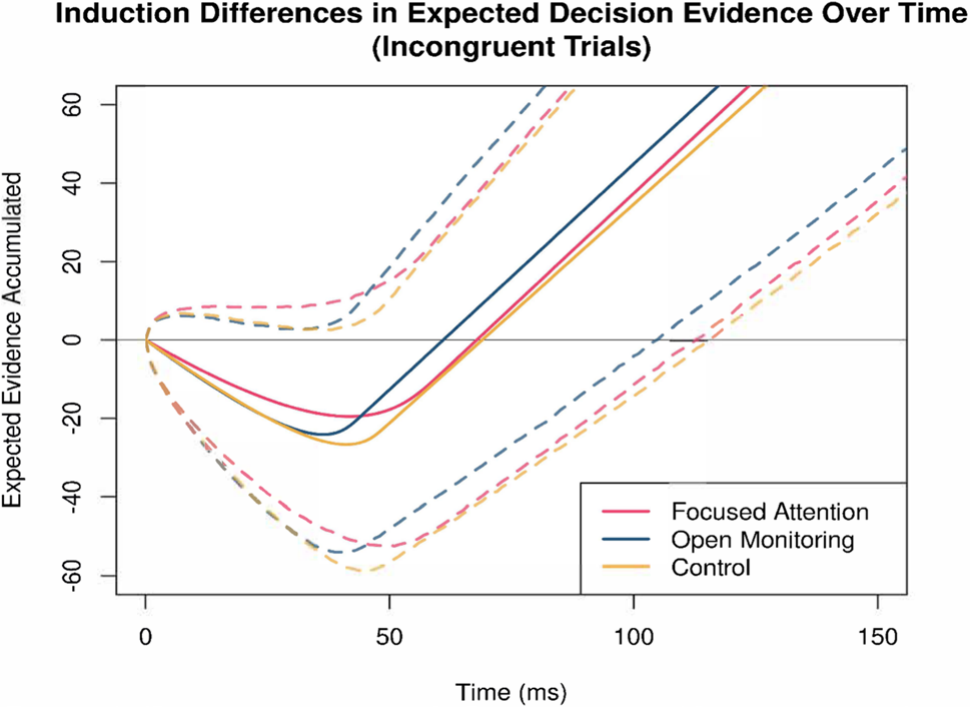
Expected attention-driven evidence accumulation by induction. *Note*. Depicted evidence accumulation time does not represent total trial response time; nondecision time must be added to produce response time. Solid lines illustrate the expected value of evidence accumulation toward a decision, quantified as drift sans noise. Dashed lines illustrate the interquartile range of 50,000 simulated trials. Under focused attention, attention was more centered on the target stimulus at the beginning of the trial (i.e., *sd*_*a*(0)_ was lower), and as a result, the focused attention condition had less of a tendency to make fast errors. In contrast, initial attentional breadth was the largest of the three conditions in open monitoring. Open monitoring, though, with the largest rate of decrease in attentional breadth at each step, was ultimately first to attend solely to the target (i.e., the first value of *t* satisfying *sd*_*a*(*t*)_ = 0.001 was lowest in the open monitoring condition). Because of that, although the open monitoring condition was more sensitive to incongruent information at the beginning of trials, it ultimately became more accurate as time went on (see also Lin et al., 2024a)

Additionally, we again observed a significant main effect of induction on the shrinkage rate parameter *r*_*d*_, *F*(2, 52) = 8.66, *p* < 0.001, *η*^2^_*p*_ = 0.250, BF_10_ = 53.39. This effect was similarly driven by the FA induction, which produced a slower rate of attentional narrowing (*M* = 0.035, *SE* = 0.007) relative to both OM (*M* = 0.080, *SE* = 0.014), *t*(26) = −3.27, *p* = 0.003, *d* = −0.74, BF_10_ = 12.85, and the active control (*M* = 0.072, *SE* = 0.013) condition, *t*(26) = −2.96, *p* = 0.007, *d* = −0.61, BF_10_ = 6.72. Although the shrinkage rate was numerically greatest in the OM condition, no statistically reliable difference in shrinkage rate was detected between the OM and active control condition, *t*(26) = 1.08, *p* = 0.291, *d* = 0.13, BF_10_ = 0.29. Figure 3 illustrates these effects.

There was also no significant main effect of induction condition on the boundary separation parameter *b*, *F*(2, 52) = 0.37, *p* = 0.694, *η*^2^_*p*_ = 0.014, BF_10_ = 0.14. There were no other significant effects of induction condition on any other model parameter, all *F* < 0.42, all *p* > 0.662, all *η*^2^_*p*_ < 0.017, all BF_10_ < 0.16. Notably, OM effects on nondecision time that were detected in the primary hierarchical analysis were not significant here, suggesting that these effects may be subtle and are thus best captured by methods that can more effectively separate induction effects from error variance—a point to which we return in the “Discussion” section to follow.

Finally, our last sensitivity analysis examined the *sd*_*a*(0)_ to *r*_*d*_ ratio. As in the hierarchical estimates, we observed a significant effect of induction condition on *sd*_*a*(0)_/*r*_*d*_, *F*(2, 52) = 4.57, *p* = 0.015, *η*^2^_*p*_ = 0.150, BF_10_ = 3.28. However, unlike in the above analyses, this effect was driven not by graded induction condition differences, but by the FA induction (*M* = 97.9, *SE* = 9.8). In particular, paralleling the *r*_*d*_ results, the FA induction showed the highest *sd*_*a*(0)_/*r*_*d*_ ratio, higher than both the OM induction (*M* = 70.6, *SE* = 7.2), *t*(26) = 2.29, *p* = 0.031, *d* = 0.65, BF_10_ = 1.85, and the active control condition (*M* = 75.7, *SE* = 1.6), *t*(26) = 2.15, *p* = 0.041, *d* = 0.53, BF_10_ = 1.46. Although the *sd*_*a*(0)_/*r*_*d*_ ratio in the OM induction condition within these estimates was numerically lower than that seen in the active control condition, this difference was not statistically reliable, *t*(26) = −0.97, *p* = 0.343, *d* = −0.12, BF_10_ = 0.26.

## Discussion

The present study leveraged computational modeling to empirically test the attentional mechanisms underlying FA and OM effects on flanker task performance. As summarized above, the behavioral findings from the parent study (Lin et al., 2024a) indicated that OM was associated with slower and more accurate responding, whereas FA did not yield reliable improvements in behavioral performance. Here, we applied an adapted SSP model to flanker data collected from a within-subject state induction design to quantify how FA and OM may differentially modulate parameters of attentional scope and decision-making. The results provided partial support for our hypotheses, and more broadly, served to advance the promise and utility of applying process-specific computational models to mindfulness research. As expected, the FA induction selectively narrowed the attentional spotlight, providing novel computational evidence for the longstanding yet relatively untested theoretical assumption that FA is characterized by reduced attentional aperture (Lutz et al., 2008, 2015). Contrary to our predictions, however, the OM induction did not produce statistically reliable modulation on parameters related to the width of visuospatial attention or decision thresholds, but was instead characterized by a faster rate of attentional narrowing coupled with longer nondecision times.

Consistent with our primary hypothesis, the FA state was distinguished by a narrower initial attentional spotlight relative to both the OM and active control conditions. This result lends quantitative support for the conceptualization of FA as a practice that narrows the scope of attention. Importantly, this pattern provides model-based evidence consistent with the intended theoretical influence of the FA induction on attentional aperture (Lutz et al., 2008, 2015), but should not be taken as definitive proof of “successful” state induction. Rather, the observed performance shift offers indirect, performance-based support for the plausibility of state transfer during task performance. Indeed, this finding highlights a pervasive methodological challenge associated with implementing state induction designs—namely, that it is often assumed but rarely demonstrated that participants successfully engage and maintain the target state during active performance. Thus, although the FA effect on the model parameter provides a promising indication of alignment between theoretical expectations and task behavior, the evidence remains inferential rather than conclusive. Moreover, despite this pattern, FA did not produce a statistically reliable reduction in behavioral flanker interference as reported in the parent study (Lin et al., 2024a). As previously suggested, one possibility, which is bolstered by our findings, is that the heightened cognitive demands required to continually sustain a restricted range of attention may detract from performance benefits for novice practitioners (Esterman & Rothlein, 2019). This idea is consistent with our finding that FA also decreased the rate of attentional narrowing, ultimately leading to relatively better performance (vs. the other two conditions) earlier in the trial but relatively worse performance later in the trial (see also Lin et al., 2024a).

In contrast, our hypothesis that OM would produce a broader attentional spotlight with a higher decision boundary was not supported by reliable evidence in the current sample. Instead, OM was unexpectedly associated with both a faster rate of attention narrowing and longer nondecision time. The nondecision time parameter indexes the perceptual encoding and motor execution components that occur outside of the evidence accumulation process (White et al., 2011). Complementing the faster shrinkage rate, the sensitivity analysis involving the *sd*_*a*(0)_/*r*_*d*_ ratio indicated that OM produced the fastest attentional convergence to the target, suggesting a uniquely efficient resolution of interference. The absence of a statistically reliable effect on spotlight width (*sd*_*a*(0)_) or decision boundary (*b*) is particularly notable, suggesting that the cautious responding observed in the parent study is not driven by the visuospatial attention mechanisms formalized by the SSP model. More critically, this inconclusive pattern implies that the “diffuse awareness” commonly attributed to OM should not be equated with a wider scope of *visual* attention. Although it remains possible that the induction failed to elicit a distinct OM state, this interpretation is not supported by the broader evidence. Manipulation check ratings indicated sufficient comprehension and comparable engagement across conditions, and the parent study demonstrated convergent induction effects distinguishing FA and OM both during the induction period (Lin et al., 2024a) and across the flanker and affective picture viewing tasks (Lin et al., 2024a, b). Accordingly, the current results are best understood as statistically inconclusive but theoretically informative, providing initial evidence to plausibly constrain the range of mechanisms through which OM may influence cognitive control. Rather than modulating the width of visuospatial attention, OM may engage fundamentally different processes wherein attention is more broadly distributed between external (e.g., flanker arrows) and internal stimuli (e.g., mental activity, bodily sensations).

Although the observed OM effects were post hoc and require cautious interpretation, they offer an alternative account for the behavioral effects. One plausible, albeit speculative, interpretation is that the longer nondecision time reflects a brief orienting period at the start of each trial. Because the OM instruction promotes awareness of internal experience, attention may not be fully primed to the external task. Consequently, upon stimulus presentation, a discrete amount of time may be required to orient and encode the flanker stimuli, thereby prolonging nondecision time, and accordingly, overall RT. This pause, coupled with a greater rate of attentional narrowing and efficient convergence, could also explain the observed increase in accuracy. By delaying the decision process until a stable perceptual representation is available, the orienting period both facilitates more rapid target selection and mitigates premature responses based on incomplete encoding. It is also possible that the interoceptive features of the OM state foster greater intentionality in motor execution, which could contribute to the longer nondecision time and reduce impulsive errors. The improvement in accuracy may therefore be a downstream consequence of a more deliberate trial-to-trial approach, rather than a direct change in the decision threshold as we had hypothesized. With that said, we reemphasize that interpretations regarding nondecision time require caution. As reported in the results, this effect was marginal in our primary analysis and was not detected by the standard observation-level method. This discrepancy may indicate that the effect of OM on nondecision time is subtle, and that its detection may require the increased sensitivity of our hierarchical modeling approach.

### Methodological Implications

The present study directly answers previous calls to leverage computational modeling within mindfulness science (van Vugt et al., 2019), showcasing the considerable promise of the approach. By moving beyond traditional behavioral measures, our application of the SSP enabled a more granular investigation to test longstanding theories involving the distinct attentional mechanisms of FA and OM. Our findings demonstrate the power of computational modeling to not only confirm or falsify process-oriented hypotheses, but also to generate new data-driven insights. Specifically, the model revealed that FA produced a narrower attentional spotlight via the *sd*_*a*(0)_ parameter, providing direct support for the theoretical alignment between this practice and the SSP’s visuospatial framework. In contrast, the model yielded an informative but statistically inconclusive result for our OM hypothesis, finding no statistically reliable effects on spotlight width (*sd*_*a*(0)_) or decision boundary (*b*) parameters, instead revealing an unexpected increase in both the rate of spotlight shrinkage and nondecision time.

By failing to detect reliable differences in decision thresholds, the model constrains the space of viable explanations for OM’s behavioral effect. This, in turn, positions the unexpected increase in the spotlight shrinkage rate (and its role in rapidly narrowing attention) and nondecision time as key parameters of interest and opens clear new directions for future investigation. Importantly, the nonsignificant contrasts observed in the present data should be interpreted as inconclusive (i.e., given Bayesian quantification of evidence from frequentist statistics, not Bayesian parameter estimation itself) rather than as evidence of equivalence or nondifference, highlighting the need for future work to explicitly evaluate such effects using more sophisticated Bayesian or equivalence-based approaches capable of quantifying support for null or near-null patterns in the data.

### Limitations and Future Research

With that said, a central limitation of this work is that the findings regarding OM were post hoc. Although the aforementioned interpretation of an orienting period is consistent with the data and broader theory, it remains fundamentally speculative and requires further direct empirical testing. Consequently, one promising avenue for future research is to conduct additional model comparisons. For example, a standard drift-diffusion model could be used to examine if an OM effect on decision boundary emerges after the SSP’s visuospatial parameters are removed. Additionally, state-switching or mixture models could be applied to formally test the proposed OM process of switching between internal and external attentional states.

Another limitation is that our results were derived from the flanker task, which primarily measures the resolution of perceptual response conflict. It is therefore unclear whether the observed findings here are specific to this domain or generalizable to other forms of conflict or facets of cognitive control. Future research should extend this modeling approach to a broader range of tasks to establish convergent and divergent validity. For instance, testing for similar effects in other conflict paradigms, such as the Stroop task or the Simon task, could clarify whether these mechanisms apply across semantic and spatial conflict. Furthermore, applying the SSP to a visual search task could assess the robustness of the FA effect on spotlight width in a different attentional context. Finally, probing other facets of cognitive control, such as response inhibition with a stop-signal task or working memory updating with the n-back task, would be likewise informative. Employing these distinct paradigms will be crucial for clarifying the boundary conditions and specific mechanisms through which FA and OM exert their influence on cognitive functioning.

Moreover, although we implemented a repeated induction structure during each testing session (i.e., presenting the same induction twice, each immediately prior to task performance), while also providing explicit reminders to maintain each state during task performance, and directly assessing condition-related differences using a post-session manipulation check, continuous verification of whether participants successfully engaged and maintained the intended mindfulness states was not possible. Indeed, despite reported comprehension, it remains fundamentally unclear whether participants were able to preserve the intended state while simultaneously processing the task stimuli. To remediate this limitation, future studies could incorporate intermittent self-report probes or leverage physiological indices (e.g., eye tracking, skin conductance) to more objectively assess state maintenance during the task performance.

Lastly, the findings here are circumscribed to a sample of mindfulness-naïve novice participants, capturing acute state effects as opposed to the effects of long-term practice. Consequently, an important avenue for future research is to apply the SSP model to a sample of experienced meditators or as part of a longitudinal training study. This would enable an informative investigation of how meditation experience or prospective training might modulate the attentional mechanisms observed here. It would be interesting, for example, to test whether expert FA practitioners exhibit an even narrower attentional spotlight or whether repeated OM training among novices also prolongs nondecision times. Collectively, these future modeling efforts will build upon the foundations established here toward a more precise and mechanistic science of mindfulness.

Although distinct mindfulness practices are known to exert differential effects on a range of neurocognitive (Brown et al., 2022; Lin et al., 2024a, b) and noncognitive outcomes, such as emotional wellbeing, stress reduction, and prosocial behaviors (Donald et al., 2019; Guendelman et al., 2017; Lindsay et al., 2018), the theoretical mechanisms thought to differentiate them remain largely untested. We addressed this gap in the present study by comparing the effects of FA and OM (vs. a control induction) on latent cognitive parameters quantified by computational cognitive modeling using a randomly assigned within-subject, crossover design. We found that FA reduced attentional spotlight width, supporting prior theories on the process and effects of this practice. In contrast, we found that OM increased both nondecision time and the rate of attentional spotlight shrinkage, though this pattern was subtle and contingent upon our hierarchical modeling approach. Together, this finding suggests that OM may foster more time to stabilize perceptual encoding prior to evidence accumulation that then facilitates target selection. In short, our results suggest that FA quite literally helps filter irrelevant information from one’s field of view, whereas open monitoring may produce a “crisper” representation of the task environment prior to engaging attentional control.

## Data Availability

All data, materials, and analytic code are publicly available on the Open Science Framework (OSF; https://osf.io/uv9yn/).

## Declarations

### Use of Artificial Intelligence

No artificial intelligence tools were used in data analysis or manuscript preparation.
